# Merging Theoretical Models and Therapy Approaches in the Context of Internet Gaming Disorder: A Personal Perspective

**DOI:** 10.3389/fpsyg.2017.01853

**Published:** 2017-10-20

**Authors:** Kimberly S. Young, Matthias Brand

**Affiliations:** ^1^Center for Internet Addiction, Russell J. Jandoli School of Journalism and Mass Communication, St. Bonaventure University, Olean, NY, United States; ^2^General Psychology: Cognition and Center for Behavioral Addiction Research, University of Duisburg-Essen, Essen, Germany; ^3^Erwin L. Hahn Institute for Magnetic Resonance Imaging, Essen, Germany

**Keywords:** internet gaming disorder, internet addiction, I-PACE model, IGD treatment

## Abstract

Although, it is not yet officially recognized as a clinical entity which is diagnosable, Internet Gaming Disorder (IGD) has been included in section III for further study in the DSM-5 by the American Psychiatric Association (APA, 2013). This is important because there is increasing evidence that people of all ages, in particular teens and young adults, are facing very real and sometimes very severe consequences in daily life resulting from an addictive use of online games. This article summarizes general aspects of IGD including diagnostic criteria and arguments for the classification as an addictive disorder including evidence from neurobiological studies. Based on previous theoretical considerations and empirical findings, this paper examines the use of one recently proposed model, the Interaction of Person-Affect-Cognition-Execution (I-PACE) model, for inspiring future research and for developing new treatment protocols for IGD. The I-PACE model is a theoretical framework that explains symptoms of Internet addiction by looking at interactions between predisposing factors, moderators, and mediators in combination with reduced executive functioning and diminished decision making. Finally, the paper discusses how current treatment protocols focusing on Cognitive-Behavioral Therapy for Internet addiction (CBT-IA) fit with the processes hypothesized in the I-PACE model.

## Introduction

Internet addiction was first identified in 1995 based on 600 case studies involving people who suffered from educational, academic, financial, or relationship problems or even job loss because they experienced a loss of control over their Internet use (Young, 1996, 1998a,b). Over the past two decades, Internet addiction research grew very quickly into a rapidly evolving field of study. Other pioneers in the field include psychologists such as Drs. David Greenfield and Marissa Hecht Orzack (Greenfield, 1999; Orzack, 1999) and Dr. Mark Griffiths (e.g., Griffiths and Hunt, 1998; Griffiths, 1999). Empirical studies started to appear with a focus on prevalence rates and psychopathological comorbidities with self-selected samples, multiple case studies, and explorations of several specific psychosocial correlates of Internet addiction, such as personality and social aspects (e.g., Armstrong et al., 2000; Morahan-Martin and Schumacher, 2000; Shapira et al., 2000; Chou, 2001; Kubey et al., 2001; Caplan, 2002). While a debated disorder, these early years of scientific research (1995–2005) created new theoretical and global models on the topic (e.g., Griffiths, 1995, 2005; Davis, 2001), which aimed at summarizing the main symptoms and potential processes underlying an excessive online activity.

In Asian cultures, the problems with dealing with Internet use are seemingly more significant compared to any other culture (potential reasons are discussed for example in Montag et al., 2016). However, in 2006, the U.S. found through a first national study that more than 10% of Americans meet at least one criterion of problematic Internet use (Aboujaoude et al., 2006). One reason for this could be that in the last 15 years, new Internet applications evolved, for example Facebook, Twitter, and WhatsApp, which make technology a significant part of most people's everyday life (Montag et al., 2015) and blur the differentiation between addictive and functional Internet use.

As early as 2008, professionals discussed the inclusion of Internet addiction in the newest version Diagnostic and Statistical Manual (DSM; Block, 2008). With an increased attention, discussion, and research, the American Psychiatric Association (APA) has recently included Internet Gaming Disorder (IGD) in section III for further study in the DSM-5 (APA, 2013). This has major effects for the research field because by listing IGD in the DSM-5 for further study, the APA hoped to encourage studies on IGD to determine whether this condition is clinically relevant and should therefore be included as a diagnosable disorder in upcoming versions of the DSM. This development was also significant and important because there is increasing evidence that people of all ages, in particular teenagers and young adults, are facing very real and sometimes very severe consequences in daily life resulting from an addictive use of online games (Young, 2004, 2015). The DSM-5 criteria include persistent use of online games, often with other players, resulting in clinically significant impairment or distress as indicated by five (or more) of the following conditions in a 12-month period:

- Preoccupation with Internet games.
- Withdrawal symptoms when Internet gaming is taken away.
- Tolerance as the need to spend increasing amounts of time engaged in Internet games.
- Unsuccessful attempts to control the participation in Internet games.
- Loss of interest in previous hobbies and entertainment as a result of, and with the exception of, Internet games.
- Continued excessive use of Internet games despite knowledge of psychological problems.
- The person has deceived family members, therapist, or others regarding the amount of Internet gaming.
- Use of Internet games to escape or relieve a negative mood (e.g., feelings of helplessness, guilt, anxiety).
- The person has jeopardized or lost a significant relationship, job, or educational or career opportunity because of participation in Internet games.

The DSM-5 notes that only online games without gambling characteristics are relevant in this proposed disorder because online gambling is included in the DSM-5 criteria for gambling disorder. Using the Internet for required activities in an educational, academic, or business context is also not included in the DSM-5 criteria for IGD. In addition, IGD does not include other recreational or social Internet use. Similarly, excessive use of Internet applications with sexual content is excluded. With moving gambling disorder to the category of substance-related and addictive disorders, the DSM-5 emphasizes parallels between substance-use disorders and behavioral addictions. With respect to Internet addiction, however, it is still discussed controversially whether the addiction concept is appropriate for describing the phenomenon. Several authors argue that a more neutral term, which does not directly imply that the behavior is addictive, would be better when referring to an uncontrolled and excessive online behavior (Kardefelt-Winther, 2014, 2017). On the other hand, there are many studies, particularly from a neuroscientific perspective, which find parallels among substance-use disorders and IGD (and also other types of Internet-use disorders) and thus justify the classification as an addiction (Weinstein et al., 2017). On a behavioral level using questionnaires, some studies, however, show that different types of behavioral addictions (i.e., gambling disorder and different types of Internet addiction) have larger overlap among them compared with the overlap among behavioral addictions and substance-use disorders (Sigerson et al., 2017), speaking for a distinct category of behavioral addictions. One has to notice, that there are also significant differences across different types of substance-use disorders (Shmulewitz et al., 2015), and they are nevertheless classified together within one category in the DSM-5. We do not go into a deep discussion of this topic here, but from our perspective, it makes sense to use the addiction concept as one framework for studying IGD and other Interne-use disorders. Naturally, it is important to additionally test alternative frameworks, for example concepts of impulse control disorders or obsessive-compulsive disorders, to better understand the real nature of IGD. Applying different theoretical frameworks to studying IGD is important since some authors argue that one problem of this research field is the lack of a theoretical background in many studies (Billieux et al., 2015; Kardefelt-Winther et al., 2017). We agree with the statement that it is important to conduct theory-driven empirical studies to contribute to a better understanding of the psychological mechanisms underlying the excessive online behavior, and we think that the addiction concept is one important framework, which can inspire theory-driven studies. The addiction concept is also helpful for creating specific treatment protocols based on the experiences within the field of substance-use disorders. We also argue that specific theoretical models of Internet-use disorders already exist (see section below), but they have to be used more intensively in empirical studies to test specific theoretical hypotheses and to increase the validity of these models. As a final note on terminology we would like to comment on the very important difference between “addicted to the Internet” and “addicted on the Internet,” which has been pointed out by Starcevic (Starcevic, 2013; Starcevic and Billieux, 2017). We agree with the perspective that the Internet is only a medium that delivers many possibilities for specific online behaviors and that it is crucial to understand the specific mechanisms underlying the different types of behaviors on the Internet. However, given that the term Internet addiction is widely used by many authors in the field, we still use this term when referring to a more general excessive online behavior. Consistent with the DSM-5 terminology, we also use the term Internet-use disorder, which should then be specified with respect to the specific online behavior (e.g., use of shopping sites, use of pornography etc.).

## The neurobiology of internet gaming disorder: a brief summary

As the scientific investigations on Internet addiction in general and IGD in specific have grown rapidly over the past 20 years, it has become very common to address neurobiological correlates of this clinical phenomenon. The knowledge about neurobiological mechanisms of IGD comprises evidence for a genetic contribution, neurochemical alterations, and both structural and functional brain correlates of IGD (Weinstein et al., 2017).

Potential genetic contributions to Internet addiction and IGD are related to the dopamine (Han et al., 2007), the serotonin (Lee et al., 2008), and the cholinergic system (Montag et al., 2012). Studies have revealed that variance of Internet addiction symptoms might be linked to genetic contributions by up to 48%, although there is also a meaningful variance across studies (Deryakulu and Ursavas, 2014; Li et al., 2014; Vink et al., 2016; Hahn et al., 2017). Results are nevertheless comparable with what is known about the genetic contribution to other psychological disorders including substance-use disorders (Egervari et al., 2017) and gambling disorder (Nautiyal et al., 2017). Genetic contributions to Internet addiction most likely interact with other psychological characteristics, such as personality, as has been shown, for example, for self-directedness (Hahn et al., 2017). Self-directedness is one of the most relevant personality traits in the context of Internet-use disorders (Sariyska et al., 2014; Gervasi et al., 2017).

With respect to brain correlates of IGD, the majority of findings show commonalities across IGD and other behavioral addictions (e.g., gambling disorder) and also substance-use disorders. A very recent comprehensive review on neuroimaging findings in IGD by Weinstein et al. (2017) emphasizes that current studies with neuroimaging techniques resemble the results of those studies on substance-use disorder (e.g., the involvement of ventral striatum as neural correlate of craving and dysfunctions in prefrontal brain areas representing deficits in inhibitory control). We here summarize some examples of neuroimaging findings, only. Gray matter density was, for example, studied by Yuan et al. (2011). They reported reduced gray matter volumes in prefrontal regions including the dorsolateral prefrontal cortex and the orbitofrontal cortex in a sample of adolescents suffering from Internet addiction. These prefrontal reductions were correlated with the addiction duration, indicating that these brain changes could reflect the reductions in inhibitory control. Inhibitory and cognitive control dysfunctions have been reported in subjects with IGD/Internet addiction, which are comparable with those found in substance-use disorders (see review in Brand et al., 2014b). Reductions in prefrontal gray matter were also reported by Weng et al. (2013), which were correlated with symptoms severity as measured by the Internet Addiction Test (Young, 1998a). On the other hand, there is also evidence for higher gray matter volume in excessive gamers, for example in the ventral striatum (Kühn et al., 2011). The higher volume of the ventral striatum may reflect a higher reward sensitivity, which has also been shown in individuals with substance-use disorders (cf. Goldstein and Volkow, 2002; Volkow et al., 2012). However, opposite findings of reduced gray matter volume of the ventral striatum have been reported recently in the context of excessive Facebook usage (Montag et al., 2017a). Given that studies in the field are not directly comparable with respect to sample constitution, study design, and analyses, more systematic research comparing different types of Internet-use disorders are necessary.

The commonalities across substance-use disorders, gambling disorder, and IGD become even more obvious when considering functional brain correlates of the disorders. One important example is the greater activity of the ventral striatum when being confronted with game-related cues (Thalemann et al., 2007; Ko et al., 2009; Ahn et al., 2015; Liu et al., 2016). This finding is also comparable with the one observed in patients with alcohol-use disorder when confronted with alcohol-related cues (e.g., Braus et al., 2001; Grüsser et al., 2004). Another example is the prefrontal cortex activity when subjects with IGD perform tasks tapping into executive functions. Prefrontal activity has been shown—dependent upon the task and prefrontal areas included in the analyses—to be both increased and decreased compared to healthy subjects (e.g., Dong et al., 2012, 2013, 2015; Brand et al., 2014b).

In summary, there is some evidence for an involvement of prefrontal and limbic brain regions in the phenomenon of IGD in particular and Internet addiction in general (cf. Kuss and Griffiths, 2012; Meng et al., 2015; Sepede et al., 2016), and—as has been shown very recently—in the addictive use of Social Networking Sites (He et al., 2017). These brain abnormalities correspond with neuropsychological functioning in IGD, especially with reduced performance in executive and cognitive control tasks (cf. Brand et al., 2014b, 2016), which are also comparable with those reported in substance-use disorders, for example in patients with alcohol-use disorder (Zhou et al., 2014). The neuropsychological findings fit with dual-process theories of addiction (cf. Bechara, 2005; Everitt and Robbins, 2016), which have recently been specified for IGD (Schiebener and Brand, 2017) and also for an addictive use of Social Networking Sites (Turel and Qahri-Saremi, 2016). The majority of neurobiological findings support the view of considering IGD as an addictive disorder, which promotes the classification in the DSM-5 category of substance-related and addictive disorders (Weinstein et al., 2017).

The challenge for the next years of neuroscientific research in the field of IGD is to show whether these brain changes are correlated with therapy success, in terms of reversibility, but also in terms of whether these brain abnormalities may predict therapy success.

## Theoretical models

Since the early case-reports 20 years ago, many studies have investigated the clinical phenomenon of Internet-use disorders, with a particular focus on IGD. As mentioned above, some authors claim that most of the clinical research on IGD and other behavioral addictions lacks a clear theoretical framework (Billieux et al., 2015; Kardefelt-Winther et al., 2017). As also outlined above, we agree with the impression that many studies that looked at psychiatric co-morbidities or personality correlates of IGD did not consider a clear theoretical background. However, we also argue that theories and theoretical models of Internet addiction already exist, which can be useful for inspiring clear hypotheses on mechanisms underlying the clinical phenomenon of IGD. The early models focused on components of Internet addictions, for example the component model by Griffiths (2005), which has been very influential, for example by inspiring the theory-driven development of assessment tools (Kuss et al., 2013). However, the components model rather summarizes the symptoms and not the psychological processes involved in Internet-use disorders. A couple of years later, two recent models of IGD or Internet addiction in general have been suggested. The model by Dong and Potenza (2014) focuses on cognitive-behavioral mechanisms of IGD and also includes some suggestions for treatment. They argue that searching for immediate reward despite long-term negative consequences plays a central role in IGD. This decision-making style is considered to interact with motivation-seeking (craving), which means both the drive to experience pleasure and the drive to reduce negative affective states. Motivation-seeking is considered to be controlled by monitoring and other executive functions and there are studies showing that inhibitory control is reduced in individuals with IGD (Argyriou et al., 2017). In their model, Dong and Potenza (2014) also included potential options for treatments. Cognitive enhancement therapy and classical cognitive-behavioral therapy are considered useful for changing the dysfunctional decision-making style and for empowering inhibitory control over motivation-seeking. Mindfulness-based stress reduction is considered to contribute to a reduction of motivation-seeking by reducing the motivation to relief from stress and negative affective states. Cognitive bias modification can influence reward sensation, which also contributes to motivation-seeking. In summary, the model by Dong and Potenza (2014) includes the interaction of cognitive (executive) components, decision-making style, and motivational components in explaining IGD, which all can principally be addressed by a combination of different treatment interventions.

Another model of IGD and Internet addiction in general has been introduced by Brand et al. (2014b). This model basically consists of three different parts (or even three different models): The first describes a functional/healthy use of the Internet, the second model aims at describing the development and maintenance of an unspecific/generalized Internet-use disorder and the third part describes potential mechanisms involved in a specific type of Interne-use disorder, for example IGD. The model of a functional use of the Internet highlights that many applications can be used for entertainment, for escaping from reality and for coping with aversive situations in daily life. However, it is argued that the functional/healthy use is characterized by the fact that the Internet is used to satisfy certain needs and goals and is stopped as soon as these goals are achieved. The second part, the model of unspecific/generalized Internet-use disorder, also considers coping mechanisms as important. However, it is assumed that a psychopathological vulnerability (e.g., depression, social anxiety) in interaction with a dysfunctional coping style and certain Internet-use expectancies explains the shift from a functional/healthy use toward an uncontrolled overuse of the Internet, without having a clear first-choice application. This perspective fits with assumptions by other researchers on a problematic use of the Internet or other media with a special focus on the role of using media for coping purposes and for escaping from reality (Kardefelt-Winther, 2014, 2017). The interaction of predisposing factors (depression, social anxiety) with the mediators dysfunctional coping and use expectancies in explaining symptoms of unspecified/generalized Internet-use disorder has been investigated using a large non-clinical sample and structural equation modeling (Brand et al., 2014a). The third part in the work by Brand et al. (2014b) aims at explaining a specific Internet-use disorder, for example IGD. In addition to the aforementioned vulnerability factors as well as dysfunctional coping and expectancies, the model suggests that specific motives for using specific applications contribute to a specific Internet-use disorder. We have additionally argued that within the addiction process, reductions of inhibitory control contribute to dysfunctional decision making with a preference for the short-term rewarding options, which results in an overuse of a specific application (see citations for studies on decision making and executive functions mentioned above).

**Figure 1:**
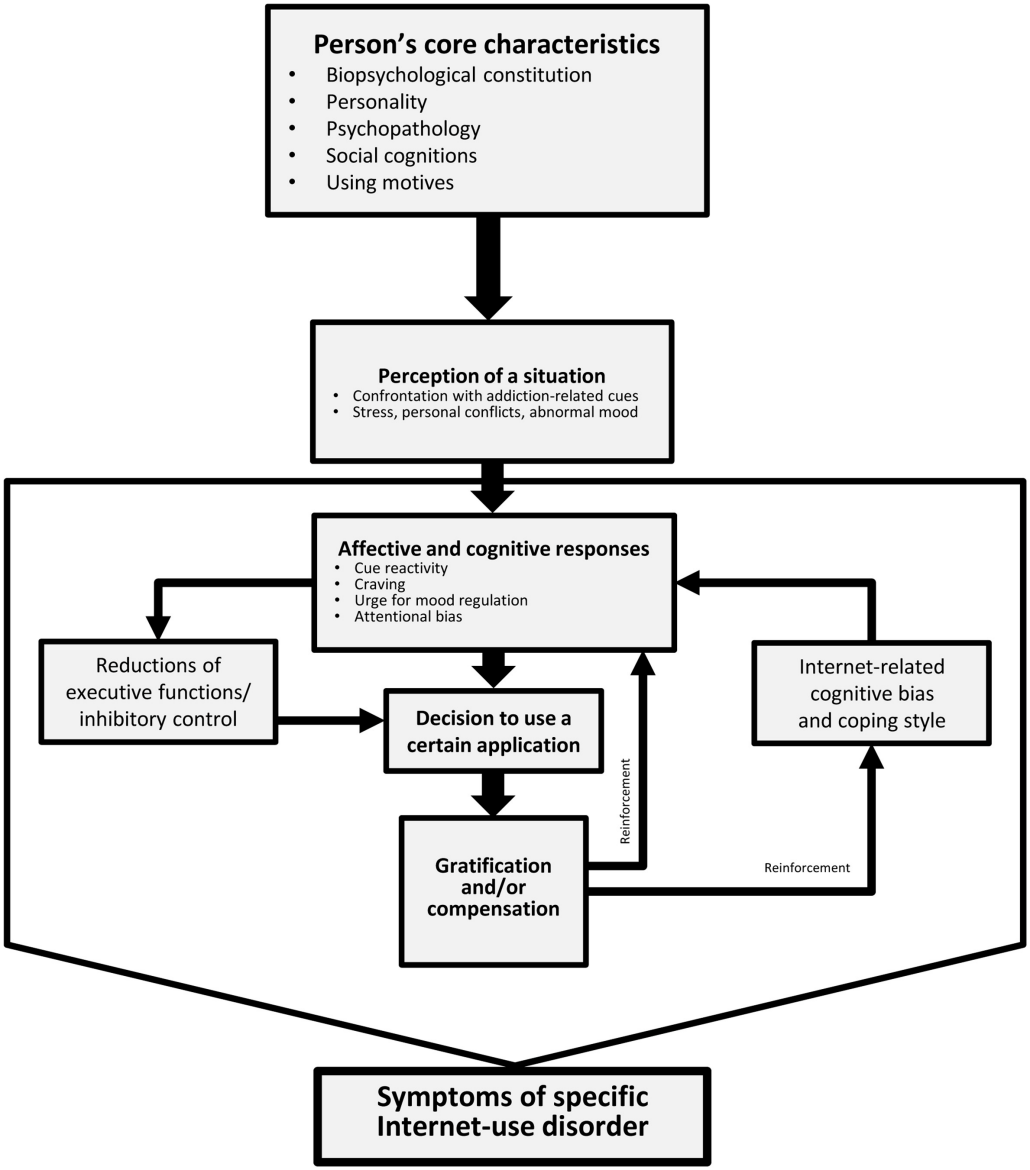
Reduced version of the I-PACE model (Brand et al., 2016).

Two years later, a revised model of specific Internet-use disorders has been suggested. Based on both new theoretical considerations and recent empirical results, the Interaction of Person-Affect-Cognition-Execution (I-PACE) model of specific Internet-use disorders was introduced (Brand et al., 2016). The I-PACE model is a theoretical framework for the hypothesized processes, which underlie the development and maintenance of an addictive use of certain Internet applications, such as gaming, gambling, pornography use, shopping, and communication. The I-PACE model is composed as a process model, including predisposing variables as well as moderator and mediator variables. Understanding the role of (changeable) moderating and mediating variables better could directly inspire therapy (see next section on treatment implications). Specific Internet-use disorders are considered to develop as a consequence of interactions between neurobiological and psychological constitutions (the predisposing variables) and moderating variables, such as coping style and Internet-related cognitive and attentional biases, as well as mediating variables, such as affective and cognitive reactions to situational triggers in combination with reduced inhibitory control. As a result of conditioning processes, these associations become stronger within the addiction process. The main interactions of a person's core characteristics (e.g., personality, psychopathology) with affective aspects (e.g., craving, motivation to experience pleasure, or to reduce negative mood), cognitive aspects (e.g., coping style, implicit positive associations), executive functions, and decision making in the course of the development and maintenance of a specific Internet-use disorder, as summarized in the I-PACE model, are illustrated in Figure 1.

The I-PACE model aims at summarizing those processes which are relevant to all types of specific Internet-use disorders. As a consequence, no gaming-specific elements have been included. Although, this is not the focus of this article, we argue that games deliver many rewards, which contributes to developing IGD on the basis of reward-conditioned cue-reactivity and craving. Many games are designed to be complex enough to be challenging and to allow players to achieve accomplishments, which keeps them playing. Both personal aspects, such as achieving goals, and social interactions, such as communicating with other players, are fundamental ingredients of many games and contribute to an “optimal experience” or feeling of flow while playing (Choi and Kim, 2004). The possibility of achieving a high score is one of the most easily recognizable hook, as players continuously try to beat the high score and this can be done endlessly in most games. In online role-playing games, players try to achieve a higher status (“level-up”), more power, and recognition by other players. Achievement, or in more detail mechanics as a sub-dimension of achievement, together with escapism were indeed clear predictors of gaming-related problems in the comprehensive study by Kuss et al. (2012). Another hook of online games is that many players create an emotional attachment to their game characters (Young, 2015). Beyond this, an important part of many games is starting or maintaining social relationships (Cole and Griffiths, 2007). Players often make friends with other players and it is these friends who may even request players to continue playing or increase the amount of time spent playing. In fact, even in ego-shooter games, most players report playing in teams. For example, in the study on personality of ego-shooter gamers by Montag et al. (2011), 90% of the 610 participants reported playing regularly as a team player. The relevance of social interactions for many gamers has also been investigated in a longitudinal study by Billieux et al. (2013). They found that discovery in combination with cooperation are the most important predictors of fast progression in online games. These results are consistent with the three-factor model (including 10 sub-factors) proposed by Yee (2006). This model suggests that achievement, social aspects, and immersion are the main components of players' motivation. This model has been examined in many studies and the main assumptions have been validated in most cases. Based on social-cognitive theory, a recent study (De Grove et al., 2016) developed a scale measuring the motivations for playing online games (or in a broader sense digital games). They also found a combination of factors including performance, social aspects, and what they call narrative (which is comparable to the discovery domain) as well as other factors (e.g., escapism, habit) being the main motivations for playing online games (see also Demetrovics et al., 2011). In summary, the most relevant motivations for playing games are achievement (or performance), social interactions, and escapism/discovery. Although, these specific motives have not been included explicitly in the I-PACE model, they represent motives for using a certain application, which is represented by “using motives” in the model and which perhaps can explain why some individuals develop IGD. Also, motives may explain why other individuals develop symptoms of Internet-pornography-use disorder, possibly because they may have a higher sexual excitability or higher trait sexual motivation (Laier et al., 2013; Laier and Brand, 2014; Stark et al., 2015). These using motives are considered person's core characteristics and are therefore important predictors of the development and maintenance of IGD or other Internet-use disorders. However, we also argue that these motives do not influence directly the development of IGD. Although, it is more likely that IGD develops in individuals who have very high gaming-related motives, the gratifications or negative reinforcements which are experienced while playing and which are consistent with the using motives accelerate the development of gaming-related implicit cognitions (e.g., attentional bias, implicit positive associations with games) and also of gaming-specific explicit use expectancies. These cognitive aspects make it more likely to develop cue-reactivity and craving in situations in which an individual is confronted with gaming-related stimuli, or in situations of negative mood or stress in daily life. These interactions of motives, the delivery of gratification feelings when playing, and changes of implicit and explicit cognitive as well as affective reactions in gaming-relevant situations are considered main processes underlying the development and maintenance of IGD (see Figure 1).

Although, the I-PACE model is hypothetical and the assumptions regarding the mechanisms which potentially underlie the development and maintenance of specific Internet-use disorders must be investigated in detail, implications for treatment can be prescribed. In the next section, we summarize some recent treatment approaches and relate them to the theoretical assumptions summarized in the I-PACE model. However, the I-PACE model only aims at explaining development and maintenance of symptoms of IGD and other Internet-use disorders. It is important to note that IGD (or generally playing computer and video games, at least if games are played without leaving the home or without physical exercise) is often linked to several further (physiological) implications, such as obesity in children and adolescents, which are related to reductions of sleep quality and overconsumption of sweet drinks (Turel et al., 2017). Such additional problems should not be neglected in therapy of IGD. However, these additional topics are not included in the I-PACE model and are therefore not addressed in the section on treatment implications.

## Treatment implications

Although, the nature of IGD and the underlying psychological mechanisms are still debated (see brief discussion in the introduction), the clinical relevance of this phenomenon is obvious. Consequently, it is necessary to provide appropriate treatment interventions to help clients to abstain from gaming or to reduce gaming behavior. In this article, we do not aim to provide a systematic review of clinical interventions of IGD, including both psychotherapy and pharmacological interventions, which can be found elsewhere (Kuss and Lopez-Fernandez, 2016; King et al., 2017; Nakayama et al., 2017).

The majority of studies have examined the use of Cognitive-Behavioral Therapy (CBT) for the treatment of Internet addiction in general or IGD in particular (Dong and Potenza, 2014; King and Delfabbro, 2014), and a first meta-analysis found that CBT outperformed other psychological treatments when referring to the time spent on online behaviors (Winkler et al., 2013).

We here concentrate on one specific type of intervention, CBT for Internet addiction (CBT-IA), and how this treatment approach relates to the I-PACE model. CBT-IA was specifically developed for treating Internet addiction by combining classical CBT elements with specific Internet-related issues (Young, 2011). CBT-IA consists of three phases: (1) Behavior modification, (2) cognitive restructuring, and (3) harm reductions. These three phases are explained in more detail within the next paragraphs. In an outcome study with 128 patients with Internet addiction (Young, 2013), CBT-IA was found to be effective in reducing symptoms, changing maladaptive cognitions, and managing underlying personal and situational factors linked to symptoms of Internet addiction. Most recently, the CBT-IA model can be applied to cases of IGD. In this case, the Internet-related elements of CBT-IA (e.g., maladaptive cognitions about the own Internet use) can be specified with respect to online games (Young, 2013).

Most consistently, treatment should first assess the client's current use of all screens and technology. Although, intake assessments are usually comprehensive and cover most relevant symptoms of psychiatric disorders including addictive behaviors, symptoms of IGD, or other types of Internet-use disorders are often overlooked in a clinical routine interview because of its newness. Some therapists are not familiar with IGD and other types of Internet addiction and may therefore overlook potential signs of this disorder. We argue that it is important that clinicians routinely assess potential symptoms of excessive and uncontrolled use of the Internet in general and IGD in specific.

With the constant availability of all Internet applications it is important to individually develop a clear and structured recovery program with each client regarding the Internet use and the use of other media or screen technology (including video games). Individuals with food addiction or binge-eating behavior evaluate part of their recovery success through objective indicators, such as the amount of caloric intake and weight loss. In analogy to this, treatment of patients with IGD should objectively measure part of the recovery success through reduced online hours, digital dieting, and abstinence from any contact with the problematic online application, which in case of IGD is the specific online game. This is what some authors refer to as digital nutrition, a concept that has been created by Jocelyn Brewer in 2013 (http://www.digitalnutrition.com.au/). Digital nutrition, however, does not mean a full abstinence from all screen technologies or Internet applications, but a healthy and functional, balanced way of using the Internet and media devices.

**Figure 2:**
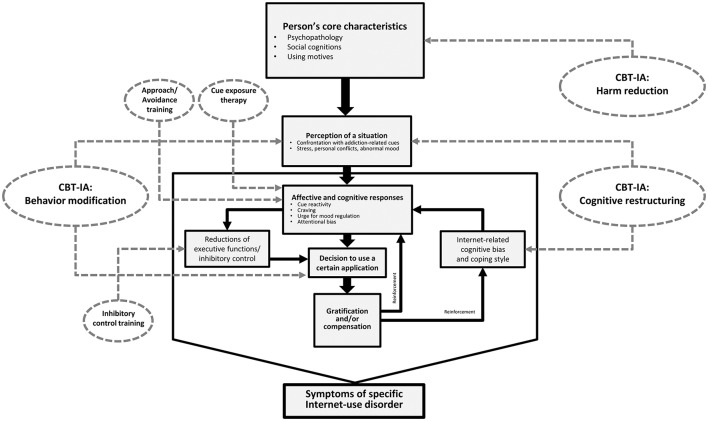
The integration of CBT-IA elements and further therapy approaches into the I-PACE model (Brand et al., 2016).

Digital nutrition is more a kind of preventive strategy for developing a healthy and functional technology use. When individuals suffer from the entire picture of IGD symptoms, therapy should help patients to abstain from gaming and to use the Internet for other purposes only moderately. This is the most difficult step, which is phase 1 of CBT-IA named behavior modification. Therapists need to monitor clients' Internet and technology use and help clients readjust contact with media and screen technology. This also means stimulus and situation control, including guiding clients change situations at home so it will become easier for them not to use the game. This can for example include computer restructuring. Subsequent behaviors become further treatment goals, for example being able to complete daily activities, maintaining a normal routine in everyday life, and spending time outside the Internet with other people (e.g., in sports or clubs) or concentrate on other hobbies. Individuals with IGD need to get re-engaged with activities that they liked prior to the game or find new activities that they can learn to love as part of abstaining from gaming. When merging the I-PACE model and CBT-IA, phase 1 of CBT-IA (behavior modification) mainly addresses the situational aspects and the decision to use a specific application (see Figure 2).

Specifically, utilizing the I-PACE model and CBT-IA model, it is important to assess a client's coping styles and Internet-related cognitive biases as well as affective and cognitive responses to the game. This is the main topic of CBT-IA phase 2: Cognitive restructuring. Individuals with IGD suffer from cognitive distortions that keep them addictively engaged in the game. For instance, they may feel lonely, restless, or even depressed but when they are playing an online game, the online character is a great warrior who feels confident and well liked. A client with low self-esteem may perceive himself as undesirable but has the impression that gaming is a way to boost his self-esteem. CBT-IA uses cognitive restructuring to break this pattern of maladaptive cognitions and Internet use expectancies (Young, 2013). “Cognitive restructuring helps put the client's cognitions and feelings “under the microscope” by challenging him or her and, in many cases, re-scripting the negative thinking that lies behind him or her” (Young, 2013, p. 210). CBT-IA can help patients with IGD understand that they are using the online game to avoid negative feelings or to escape from reality and that they are thinking they receive more positive feelings when playing the game compared to any other activity in daily life. This is sometimes hard for the clients, but it is important for therapy success to understand and change these maladaptive thoughts. Again, the focus of both the I-PACE and CBT-IA model is to examine the mechanisms of experiencing gratification by playing the game and also the needs which are not satisfied in real life and which are compensated by playing excessively (Young, 2013; Brand et al., 2016).

Cognitive restructuring with clients is also useful for helping the clients with IGD re-evaluate how rational and valid his or her interpretations of situations and feelings are. For instance, a client who uses online games as a way to feel better about his or her life and to feel strong, powerful, and well recognized will begin to realize that he or she is using the online game to satisfy needs that are unsatisfied in his or her real life. In this context, CBT-IA helps the client to develop more functional and healthy coping strategies to deal with real life stress and negative feelings and to find healthy ways to increase self-esteem and self-efficacy and to build stable interpersonal relationships.

As in many addictions, the most common response in players who do see they have a problem with online gaming is a “guilt-and-purge cycle.” True recovery, at least for most gamers, involves looking at the motives and expectancies underlying the game habit. Treatment must also help clients recognize, address, and treat the underlying issues co-occurring with IGD, which is the main aspect of CBT-IA phase 3: Harm reduction. Particularly, underlying depression and social anxiety should be treated.

CBT-IA can be complemented by recently suggested neurocognitive trainings, which have been evaluated positively in the context of substance-use disorders. One example is a retraining of implicit cognitions, which can potentially result in avoidance rather approach tendencies when experiencing craving (Wiers et al., 2011; Eberl et al., 2013a,b). Attentional retraining programs (e.g., Schoenmakers et al., 2010; Christiansen et al., 2015) may be useful to increase clients' inhibitory control (e.g., Houben and Jansen, 2011; Houben et al., 2011; Bowley et al., 2013). This could be done for example by using Go/No-Go Tasks with addiction-related stimuli. However, future studies must demonstrate that these techniques are helpful for increasing inhibitory control in the context of IGD. Cue-exposure therapy (Park et al., 2015) can be useful for reducing the intensity of experienced craving (Pericot-Valverde et al., 2015), which is consistent with current neuroimaging findings in IGD (Zhang et al., 2016).

The synthesis of the I-PACE model's main assumptions about potential processes involved in the development and maintenance of IGD and other Internet-use disorders and some of the most relevant therapy techniques (CBT-IA and additional approaches) is illustrated in Figure 2. Although, this figure concentrates on the I-PACE model, it also fits widely with assumptions raised by other authors (Dong and Potenza, 2014). As outlined above, in their model, Dong and Potenza (2014) argued that cognitive behavioral therapy and cognitive enhancement therapy are useful for changing the decision-making style and for increasing inhibitory control over the motivation to use online games. Cognitive bias modification, which is comparable to what is called cognitive restructuring in CBT-IA, is helpful for influencing clients' expectancies to experience reward when playing the game (Zhou et al., 2012). Future studies should also investigate in how far the medium Internet itself is useful for helping clients. Some very recent research focuses on Apps that guide clients through daily life and that help them to reduce stress (e.g., by mindfulness-based stress reduction) or to better deal with negative mood, but such Apps can also track the client's time spent online, which can also be useful for therapy. A recent summary of the contributions of psychoinformatics to the treatment of Internet addiction can be found in Montag et al. (2017b).

**Figure 3:**
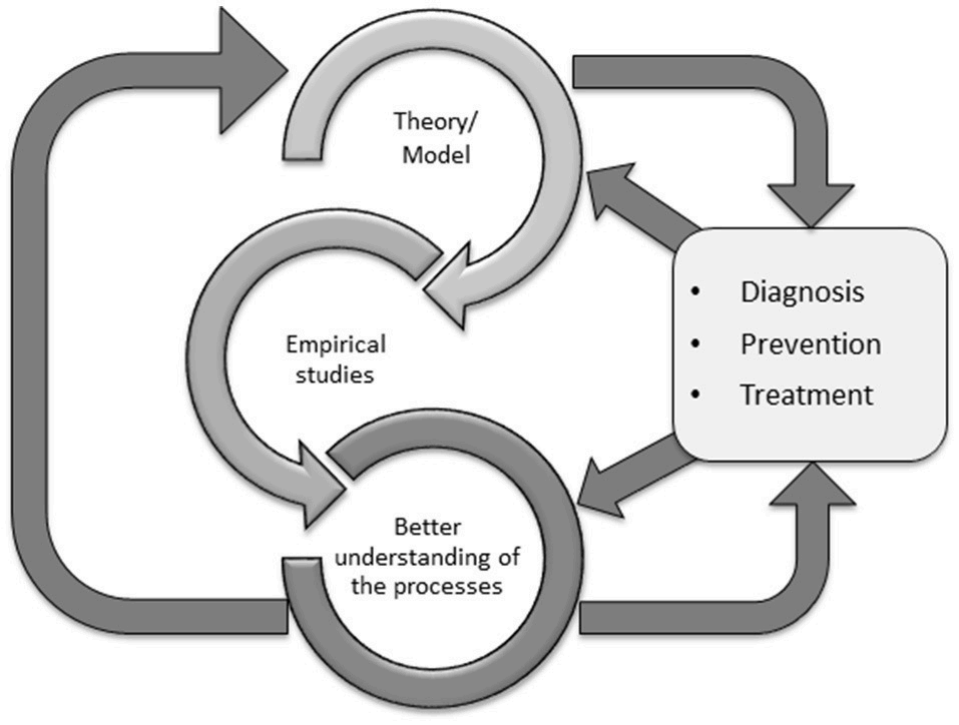
The bidirectional relationship between theoretical models and clinical practice.

Why is it helpful to merge theoretical models of Internet-use disorders (such as the I-PACE) and existing therapy approaches (such as CBT-IA) for both research and clinical practice? We argue that theoretical models have the goal to summarize the main processes underlying both the development and the maintenance of a disorder. These models are useful for specifying research hypotheses on the assumed processes. If we then understand better the core processes involved in the phenomenology of a disorder, we can check whether these processes are addressed by existing therapy approaches, and if not, how current treatment protocols can be complemented by additional specific techniques. On the other hand, studies on the efficacy of treatment approaches can also inspire theoretical models of the disorder. If we see for example that cognitive restructuring is particularly helpful for the clients then obviously cognitive processes (e.g., expectancies) are specifically important in the maintenance of the disorder, and existing models can be checked if they have considered these processes adequately. In summary, the relationship between theoretical models and therapy is bidirectional. This relationship is summarized in Figure 3.

When merging the I-PACE and the CBT-IA model, we see that the three main phases of CBT-IA address particularly those variables which are considered moderating and mediating variables in the I-PACE model. We see, however, that most likely CBT-IA can be complemented by additional techniques (smaller ellipses in Figure 2). Both the I-PACE and CBT-IA model are also useful for developing new assessment tools for clinical practice. For example, if we see in empirical studies that Internet-use expectancies are critically involved in explaining symptoms of Internet-use disorders (Brand et al., 2014a) and we see that cognitive restructuring is useful for changing these expectancies (Young, 2013), it would be helpful to have validated tools assessing Internet-use expectancies for clinical practice. It would also be helpful to include this issue in prevention programs. Figure 3 aims at summarizing the bidirectional relationships between theories (and consequently empirical studies on processes) and clinical practice including diagnosis, prevention, and therapy. Given that both theoretical models and therapy approaches (and also diagnosis and prevention) are never final or perfect, it is important to consider how these two areas can successfully interact and influence each other to increase validity and efficacy.

## Conclusions

This paper reviews the most relevant neurobiological studies associated with the development of IGD, some theoretical models of the development and maintenance of IGD and other specific Internet-use disorders, and treatment implications for addicted clients using the I-PACE and CBT-IA models.

Current neuroimaging studies indicate that IGD and other behavioral addictions (e.g., gambling disorder) as well as substance-use disorders share several similarities. Similarities can be seen on the molecular level (e.g., genetic contribution), neurocircuitry (e.g., the dopamine fronto-striatal loops including ventral striatum and several parts of the prefrontal cortex), and behavioral levels including implicit (e.g., attentional bias) and explicit emotions and cognitions (Brand et al., 2016). As we move forward, the diagnosis of IGD has several implications from the clinical, educational, and cultural contexts.

Clinically, more attention and training should be applied in counseling training, schools, and institutions. Given its newness, symptoms of IGD are still overlooked by some clinicians. Therefore, it is important that clinicians are trained in assessment procedures and routinely check for the presence of excessive and uncontrolled Internet use in their practices. In addition, clinicians should be trained in treatment of IGD and other types of Internet-use disorder. Treatment protocols must be further studied and improved. Indeed, while early outcome data show CBT-IA offers an effective approach to helping clients maintain a healthy online routine, further studies should examine other therapeutic modalities such as group therapy, family therapy, and *in vivo* counseling to look at their combined treatment efficacy.

If indeed IGD is viewed as a disorder this would also have implications for school systems to develop screen smart policies that protect children and adolescents from developing IGD problems. It would be helpful to have educators receive training on how to identify students who are most at-risk for developing IGD. It would be helpful for school administrators to develop policies for technology use by students in the classrooms in order to prevent IGD from occurring, strategies may include limited screen use in the classroom, no gaming policies, and encouragement of social clubs at school.

On the other hand, it makes also sense to note that there are several limitations of the current state-of-the-art in IGD research. There is an ongoing debate about classification, diagnostic criteria and instruments, conceptualization as an addiction or another type of disorder, and many other unsolved problems or challenges for the research aiming at understanding the nature of IGD and other Internet-use disorders. Consequently, it is mandatory not to overpathologize a healthy and balanced use of the Internet in general or games in particular, as long as the use is integrated in daily life without experiencing severe negative consequences.

Theoretical models can inspire empirical studies investigating the nature of IGD and other Internet-use disorders. It is important to use those models for spelling out clear research hypotheses in future studies. Both congruent and divergent validities should be addressed systematically in future studies. Although, the theoretical background for the I-PACE model is the addiction framework, we also have to consider other theoretical approaches within empirical studies to contribute to a better understanding of the underlying mechanisms. Future studies will demonstrate which aspects of the addiction framework and which parts of other theories are valid in explaining IGD. Theoretical models on a disorder can potentially inspire therapy approaches, but only if these theoretical models are valid and have been tested empirically. One of the important challenges for future IGD research is to merge existing theoretical assumptions about the underlying psychological mechanisms of the disorder with therapy and prevention techniques. The inspirations of theory and therapy should be bidirectional and in the best case, research on psychological mechanisms and therapy research interact in concert.

## Author contributions

All authors listed have made a substantial, direct and intellectual contribution to the work, and approved it for publication.

### Conflict of interest statement

The authors declare that the research was conducted in the absence of any commercial or financial relationships that could be construed as a potential conflict of interest.
